# The complete chloroplast genome of *Trachyspermum ammi* reveals a species-specific inverted repeat expansion

**DOI:** 10.3389/fpls.2026.1837075

**Published:** 2026-07-13

**Authors:** Ramawatar Nagar, Vandna Patial, Nimmy M. S., Sharda Choudhary

**Affiliations:** 1 ICAR-National Institute for Plant Biotechnology, New Delhi, India; 2 Division of Biochemistry, ICAR-Indian Agricultural Research Institute, New Delhi, India; 3 ICAR-National Research Centre on Seed Spices, Ajmer, India

**Keywords:** ajwain, *Trachyspermum ammi*, Apiaceae, Apioideae, chloroplast genome, inverted repeat expansion, seed spice crop, plastome

## 1 Introduction

*Trachyspermum ammi* (L.) Sprague, commonly known as ajwain or carom, is an aromatic annual herb in the family Apiaceae, subfamily Apioideae, and one of the most commercially significant seed spice crops of South Asia and the Middle East (Bairwa et al., 2012). The plant is native to Egypt and the eastern Mediterranean, and is extensively cultivated across the arid and semi-arid regions of India (particularly Gujarat and Rajasthan), Pakistan, Iran, Afghanistan, and Iraq (Asif et al., 2014; Siddiquie et al., 2024). *T. ammi* produces characteristic ovoid, greyish-brown mericarps (commonly called seeds) that are highly valued in Indian culinary traditions and across Ayurvedic, Siddha, and Unani systems of medicine (Bairwa et al., 2012; Hanif et al., 2021). The fruits possess a pungent, thyme-like aroma attributable to essential oils dominated by thymol, a monoterpene phenol, alongside γ-terpinene, p-cymene, and other volatile compounds (Soltani Howyzeh et al., 2018). Ethnobotanically, ajwain is used for the treatment of digestive disorders (flatulence, indigestion, dyspepsia), respiratory ailments (asthma, bronchial conditions), abdominal pain, and gynaecological complaints, among other conditions (Bairwa et al., 2012; Hanif et al., 2021; Siddiquie et al., 2024).

Despite the considerable economic, medicinal, and cultural importance of *T. ammi*, its genomic resources remain limited. Previous molecular studies have been largely restricted to transcriptome-based investigations of thymol biosynthesis (Soltani Howyzeh et al., 2018), inflorescence development (Amiripour et al., 2019), and the identification of SSR molecular markers (Soltani Howyzeh et al., 2019). The complete chloroplast genome sequence of *T. ammi* has not been fully characterized to date. A ~154 kb *T. ammi* chloroplast genome (NC_047246.1) is available in NCBI and has been reported as complete. This may represent a partial or differently assembled genome, as it is notably smaller (~154 kb) than our assembly and the independently sequenced *T. ammi* accession PV794607.

Chloroplast genomes (plastomes) in angiosperms are typically circular, quadripartite molecules of 120–170 kb, encoding 120–130 genes encompassing components of the photosynthetic apparatus, ribosomal proteins, RNA polymerase subunits, ribosomal RNAs, and transfer RNAs (Cauz-Santos, 2025; Daniell et al., 2016). The canonical quadripartite architecture comprises a large single-copy (LSC) region and a small single-copy (SSC) region separated by a pair of inverted repeats (IRA and IRB). Their slow evolutionary rate, maternal inheritance, low recombination frequency, and largely conserved gene order have established plastomes as primary markers for phylogenetic reconstruction and species-level barcoding across angiosperms (Downie et al., 2010; Fan et al., 2021; Wen et al., 2021).

Within Apiaceae, subfamily Apioideae is exceptional among angiosperms for the frequency and magnitude of structural variation in the plastome, particularly dynamic shifts in the inverted repeat (IR) boundaries (Cauz-Santos, 2025). Plunkett and Downie (2000) documented at least ten independent expansions and contractions of the IR across Apioideae, with boundary shifts ranging from ~1 to 16 kb. Genera such as *Angelica*, *Ferula*, *Peucedanum*, *Melanosciadium*, *Ligusticum*, *Sanicula*, *Glehnia*, and *Cnidium* have all been reported to exhibit significant IR boundary polymorphism (Downie et al., 2010; Wen et al., 2021). These IR boundary shifts are consequential because they alter gene copy number; genes transferred from the single-copy LSC into the duplicated IR are subject to IR-mediated gene conversion, which tends to homogenise the two IR copies and may reduce substitution rates in the captured genes (Plunkett and Downie, 2000).

In the present study, we report the complete chloroplast genome of *T. ammi* cv. AA-1 and compare with those of *T. scaberulum* (NC_070346) and *T. triradiatum* (PV794612) to characterise the structural basis of the observed genome size difference and assess its evolutionary significance within the genus. This resource provides a foundational genomic reference for future research on *T. ammi* genetics, molecular breeding, pharmacological enhancement, and phylogenomics of Apioideae.

## 2 Materials and methods

### 2.1 Plant material and DNA extraction

Seeds of *Trachyspermum ammi* cultivar AA-1 were surface-sterilized and germinated on moistened filter paper at 25 ± 2 °C under a 16 h light/8 h dark photoperiod. Young leaves were harvested from two-week-old seedlings, immediately snap-frozen in liquid nitrogen, and stored at −80 °C until use. Total genomic DNA was extracted following a modified cetyltrimethylammonium bromide (CTAB) protocol (Nagar and Schwessinger, 2018). DNA quality was assessed by measuring the A_260_/A_280_ absorbance ratio on a NanoDrop 2000 spectrophotometer (Thermo Fisher Scientific, USA) and by 1% agarose gel electrophoresis. Only samples with an A_260_/A_280_ ratio of 1.8–2.0 and a minimum yield of 1.5 μg were used for library preparation.

### 2.2 Library preparation and sequencing

Sequencing libraries were constructed from high-quality genomic DNA using the NEBNext Ultra II DNA Library Prep Kit for Illumina (New England Biolabs, UK) according to the manufacturer’s instructions, incorporating end-repair, dA-tailing, adapter ligation, and PCR enrichment steps. Paired-end sequencing (2 × 150 bp) was performed on the Illumina NovaSeq 6000 platform (Illumina, USA) at a commercial sequencing facility.

### 2.3 Chloroplast genome assembly and annotation

Raw FASTQ reads were quality-assessed using FastQC v0.11.9 (Andrews, 2010; https://www.bioinformatics.babraham.ac.uk/projects/fastqc/). Adapter sequences and low-quality bases (Phred score < 20) were removed using Trim Galore v0.6.7 (Krueger, 2015; https://www.bioinformatics.babraham.ac.uk/projects/trim_galore/) with default parameters. Chloroplast reads were assembled *de novo* using GetOrganelle v1.7.5 (Jin et al., 2020). Assembly graph circularity and completeness were verified using Bandage v0.8.1 (Wick et al., 2015). Genome annotation was performed using three complementary tools to maximise accuracy: GeSeq v2.03 (Tillich et al., 2017) with default eudicot reference sets, CPGAVAS2 (Shi et al., 2019), and PGA (Qu et al., 2019). Annotation conflicts between the three tools were resolved by manual inspection, with particular attention to start and stop codon positions, CDS reading frames, intron–exon boundaries, and gene strand orientations. The final annotated sequence was visualized as a circular map using CPGView (Liu et al., 2023). The complete annotated chloroplast genome sequence of *T. ammi* cv. AA-1 has been deposited in the NCBI GenBank database under accession number PZ091433; raw whole-genome sequencing reads are available under BioProject ID PRJNA1437723.

### 2.4 Comparative analysis of inverted repeat boundaries

To investigate inverted repeat contraction and expansion of the chloroplast genome of *T*. *ammi*, the boundaries of the large single-copy (LSC) region, small single-copy (SSC) region, and the two IR regions (IRA and IRB) were compared across four *Trachyspermum* plastomes: *T. ammi* cv. AA-1 (PZ091433, this study), *T. ammi* isolate YL11021 (PV794607), *T. scaberulum* (NC_070346), and *T. triradiatum* isolate YL09151 (PV794612). To ensure consistency, all the genomes were reoriented relative to the *rbcL* gene using the Rotate v1.0 tool (Durbin et al., 2023) and subsequently reannotated using GeSeq v2.06 (Tillich et al., 2017). IR boundary positions were determined from the annotated repeat region features and confirmed by examining the positions of known boundary-flanking genes (*rpoA* at the LSC/IRB junction; *ycf1* at the IRB/SSC junction; *ndhF* in the SSC; *rpl2* and *rpl23* at the IRA/LSC junction). The IR boundary positions and junction gene configurations of all four genomes were visualized using CPJSdraw v1.0.0 (Li et al., 2023).

## 3 Preliminary data analysis

**Figure 1:**
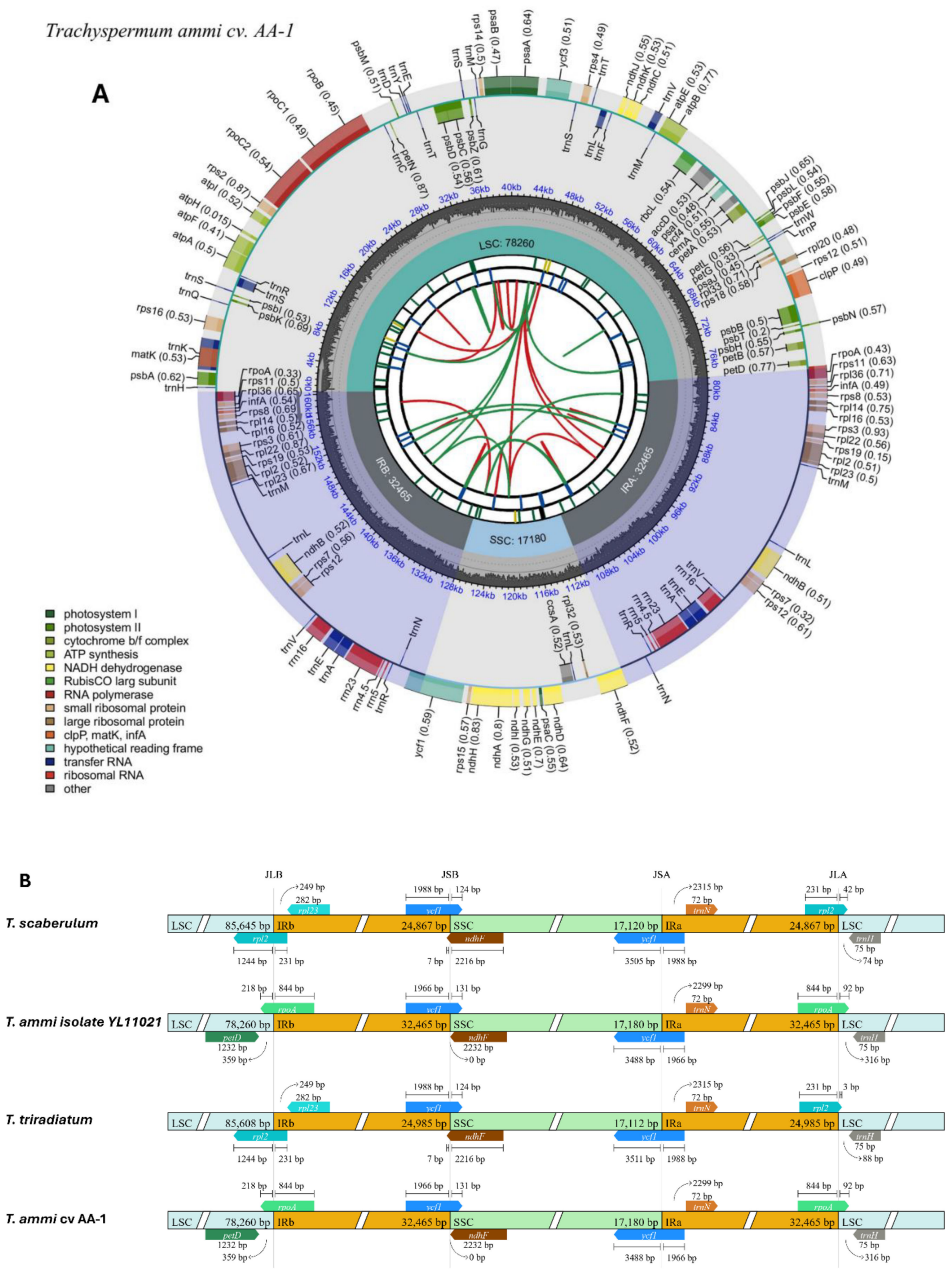
Chloroplast genome structure of *Trachyspermum ammi* cv. AA-1. **(A)** Circular map of the complete chloroplast genome (160,370 bp) showing the typical quadripartite structure with the large single-copy (LSC, 78,260 bp), small single-copy (SSC, 17,180 bp), and two inverted repeats (IRA and IRB, each 32,465 bp; highlighted). Genes on the inner face of the ring are encoded on the negative strand (transcribed clockwise); those on the outer face are on the positive strand (transcribed counterclockwise). The innermost gray histogram tracks GC content variation around the genome. **(B)** Comparative analysis of the inverted repeat (IR) boundary junctions among *T. ammi* (cv. AA-1 and isolate YL11021), *T*. *scaberulum*, and *T*. *triradiatum*. The four IR/SC/LSC junctions are shown: JLB (LSC/IRB), JSB (IRB/SSC), JSA (SSC/IRA), and JLA (IRA/LSC). Orange bars represent the IR regions, while adjacent segments represent the LSC and SSC regions. Numbers indicate the distance (bp) from each junction to the nearest gene. Red arrows highlight the species-specific IR expansion (~7,321 bp per copy) in *T. ammi* at the LSC/IRB junction.

The complete chloroplast genome of *T. ammi* cv. AA-1 (GenBank accession PZ091433) is a circular molecule of 160,370 bp (**Figure 1A**). It exhibits the typical quadripartite structure consisting of a large single-copy (LSC) region of 78,260 bp, a small single-copy (SSC) region of 17,180 bp, and a pair of inverted repeats (IRA and IRB) of 32,465 bp each. The plastome of *T. ammi* is larger than those typically reported for most Apiaceae (Huang et al., 2022; Ruhlman et al., 2006). This increased genome size is largely attributable to the species-specific expansion of the IR, a type of structural variation extensively documented in Apiaceae (Plunkett and Downie, 2000). The overall GC content is 37.73%, which falls within the range commonly reported for eudicot plastomes (Daniell et al., 2016; Ruhlman et al., 2006). As expected, GC content varies across regions, being highest in the IR (41.41%), moderate in the LSC (36.04%), and lowest in the SSC (31.50%). These patterns are consistent with those observed in other members of the Apiaceae family (Huang et al., 2022). Genome annotation revealed 113 unique genes, including 79 protein-coding genes, 30 tRNA genes, and 4 rRNA genes (**Table 1**). This gene content is typical for the Apiaceae subfamily Apioideae (Huang et al., 2022; Wang et al., 2021). Fifteen protein-coding genes and six tRNA genes contain introns. Among them, *clpP* and *ycf3* each harbour two introns, while *atpF*, *rpoC1*, *rpl2*, *rpl16*, *petB*, *petD*, *rps16*, *ndhA*, and *ndhB* contain one intron each. The maturase gene *matK* is nested within a large group IIA intron of *trnK-UUU*, a conserved structural arrangement found across angiosperm plastomes. The *rps12* gene is trans-spliced, with its 5′ exon located in the LSC and the 3′ exons duplicated in the IR regions (Ruhlman et al., 2006).

**Table 1:** Gene composition of the chloroplast genome of *Trachyspermum ammi* cv. AA-1.

Function	Gene group	Genes
Photosynthesis		
Photosynthesis	Photosystem I (5)	*psaA*, *psaB*, *psaC*, *psaI*, *psaJ*
	Photosystem II (15)	*psbA*, *psbB*, *psbC*, *psbD*, *psbE*, *psbF*, *psbH*, *psbI*, *psbJ*, *psbK*, *psbL*, *psbM*, *psbN*, *psbT*, *psbZ*
	F-type ATP synthase (6)	*atpA*, *atpB*, *atpE*, *atpF*^b^, *atpH*, *atpI*
	NADH dehydrogenase (11)	*ndhA*^b^, *ndhB*^ba^, *ndhC*, *ndhD*, *ndhE*, *ndhF*, *ndhG*, *ndhH*, *ndhI*, *ndhJ*, *ndhK*
	Cytochrome b_6_/f complex (6)	*petA*, *petB*^b^, *petD*^b^, *petG*, *petL*, *petN*
	Large subunit of RuBisCO (1)	*rbcL*
Genetic apparatus		
Transcription	RNA polymerase (4)	*rpoA*^a^, *rpoB*, *rpoC1*^b^, *rpoC2*
Ribosomal proteins	Large subunit (9)	*rpl2*^ba^, *rpl14*^a^, *rpl16*^b^, *rpl20*, *rpl22*^a^, *rpl23*^a^, *rpl32*, *rpl33*, *rpl36*^a^
	Small subunit (13)	*rps2*, *rps3*^a^, *rps4*, *rps7*^a^, *rps8*^a^, *rps11*^a^, *rps12*^da^, *rps14*, *rps15*, *rps16*^b^, *rps18*, *rps19*^a^
	Translation initiation factor (1)	*infA* ^a^
Structural RNAs		
Structural RNAs	Transfer RNAs (30)	*trnA-UGC*^ba^, *trnC-GCA*, *trnD-GUC*, *trnE-UUC*, *trnF-GAA*, *trnG-GCC*, *trnG-UCC*^b^, *trnH-GUG*, *trnI-CAU*^a^, *trnI-GAU*^ba^, *trnK-UUU*^b^, *trnL-CAA*^a^, *trnL-UAA*^b^, *trnL-UAG*, *trnM-CAU*, *trnN-GUU*^a^, *trnP-UGG*, *trnQ-UUG*, *trnR-ACG*^a^, *trnR-UCU*, *trnS-GCU*, *trnS-GGA*, *trnS-UGA*, *trnT-GGU*, *trnT-UGU*, *trnV-GAC*^a^, *trnV-UAC*^b^, *trnW-CCA*, *trnY-GUA*, *trnfM-CAU*
	Ribosomal RNAs (4)	*rrn4.5*^a^, *rrn5*^a^, *rrn16*^a^, *rrn23*^a^
Other functions		
Post-transcriptional modification	Maturase (1)	*matK*
Other	ATP-dependent protease (1)	*clpP* ^c^
	Acetyl-CoA carboxylase (1)	*accD*
	Envelope membrane protein (1)	*cemA*
	Cytochrome c haem attachment (1)	*ccsA*
Genes of unknown or hypothetical function		
Unknown/hypothetical	Hypothetical chloroplast reading frames (3)	*ycf1*, *ycf2^a^, ycf3^c^*, *ycf15*^a^

a Genes duplicated in both inverted repeat regions (IRA and IRB).

b Gene contains one intron.

c Gene contains two introns.

d Gene is trans-spliced; the 5′ exon of rps12 is in the LSC and the duplicated 3′ exons are in the IR regions.

The most distinctive feature of the *T. ammi* plastome is a pronounced species-specific expansion of the inverted repeat. This IR expansion is consistent across two independent *T. ammi* accessions (PZ091433 and PV794607) that share 100% sequence identity, confirming that it is a stable species level characteristic rather than an assembly artefact. Each IR measures 32,465 bp, which is ~7,598 bp larger than those of its congeners, *T*. *scaberulum* (24,867 bp) and *T*. *triradiatum* (24,985 bp). Consequently, the IR regions constitute 40.5% of the total *T. ammi* genome, compared with 32.6% in *T*. *scaberulum* and 32.7% in *T*. *triradiatum*.

At 32,465 bp, the IR of *T. ammi* is among the largest reported in the subfamily Apioideae. Similar IR sizes have been reported in *Ferula sinkiangensis*, *Melanosciadium pimpinelloideum*, and other *Ferula* species, whereas even larger IRs occur in the long-type plastome of *Peucedanum japonicum* (Fan et al., 2021; Joh et al., 2025; Lee et al., 2019; Tan, 2020; Yang et al., 2022). However, unlike *Ferula* and *Angelica*, where large IRs are shared across multiple species and likely represent ancestral expansions, the enlarged IR of *T. ammi* appears to be species-specific. Both sequenced congeners retain the typical Apioideae IR size of approximately 25 kb, suggesting that the expansion occurred after the divergence of *T. ammi* from the common ancestor of the genus.

Comparative analysis of the IR boundaries (**Figure 1B**) shows that the LSC/IRB junction in *T. ammi* has shifted inward by ~7,321 bp into the LSC region compared to other species of the *Trachyspermum* genus. As a result, the RNA polymerase gene *rpoA* now straddles the new junction, with only its 5′ end (218 bp) remaining in the LSC. This boundary shift has led to the duplication of 10 ribosomal protein genes (*rps11*, *rpl36*, *infA*, *rps8*, *rpl14*, *rpl16*, *rps3*, *rpl22*, *rps19*, and *rpl2*), which remain single copy in the LSC of *T*. *scaberulum* and *T*. *triradiatum*.

The molecular mechanism most widely accepted to explain IR boundary shifts in angiosperm plastomes is illegitimate recombination between short dispersed repeats located near the IR/single-copy junctions, followed by IR-mediated gene conversion that progressively extends one IR copy at the expense of the adjacent single-copy region (Cauz-Santos, 2025; Plunkett and Downie, 2000). The IR expansion observed in *T. ammi* is consistent with this model and appears to be restricted to the LSC/IRB boundary. In contrast, the SSC region remains highly conserved among the three examined *Trachyspermum* species, measuring 17,180 bp in *T. ammi*, 17,120 bp in *T. scaberulum*, and 17,112 bp in *T. triradiatum*. Likewise, the IRB/SSC boundary gene *ycf1* exhibits an identical arrangement in all three species. Such asymmetric expansion affecting only the LSC/IR boundary is characteristic of IR expansion events documented across Apioideae (Plunkett and Downie, 2000; Wang et al., 2021).

A major consequence of this expansion is the duplication of ten ribosomal protein genes (*rps11, rpl36, infA, rps8, rpl14, rpl16, rps3, rpl22, rps19*, and *rpl2*) that were ancestrally located in the LSC. These genes are now incorporated into the duplicated IR and therefore exist as two identical copies. Genes located within IR regions are subject to continuous copy correction through IR-mediated gene conversion, a process that suppresses mutation accumulation and maintains sequence identity between the two IR copies (Birky-Jr. and Walsh, 1992; Wolfe et al., 1987; Zhu et al., 2016). Across land plants, synonymous substitution rates in IR-located genes are approximately 3.7-fold lower than those of genes residing in single-copy regions (Zhu et al., 2016). Accordingly, the duplicated ribosomal protein genes in *T. ammi* are expected to experience stronger purifying selection and maintain higher sequence fidelity than their homologues in *T. scaberulum* and *T. triradiatum*.

The enlarged IR, together with the unique boundary configuration involving *rpoA* and the duplicated ribosomal protein genes, represents a useful molecular character for species identification and phylogenetic studies within *Trachyspermum* genus. Further sampling of additional species will be necessary to determine whether this structural rearrangement is unique to *T. ammi* or shared with closely related taxa within the tribe Ammineae.

## Data Availability

The datasets presented in this study can be found in the NCBI (https://www.ncbi.nlm.nih.gov). The annotated chloroplast genome sequence is available in the GenBank under accession number PZ091433, and the raw sequencing data are available under BioProject ID PRJNA1437723.
